# Intraoperative Guidance of Pancreatic Cancer Resection Using a Toll-like Receptor 2–Targeted Fluorescence Molecular Imaging Agent

**DOI:** 10.1158/2767-9764.CRC-24-0244

**Published:** 2024-11-05

**Authors:** Amanda S. Huynh, Allison S. Cohen, Michael Doligalski, Todd J. Casagni, Valerie E. Moberg, Xuan Huang, Jennifer Morse, Dominique Abrahams, Mark C. Lloyd, Barbara A. Centeno, Margaret K. Baldwin, Mark L. McLaughlin, Josef Vagner, David L. Morse

**Affiliations:** 1 Department of Metabolism and Physiology, H. Lee Moffitt Cancer Center & Research Institute, Tampa, Florida.; 2 Department of Comparative Medicine, H. Lee Moffitt Cancer Center & Research Institute, Tampa, Florida.; 3 Analytic Microscopy Core, H. Lee Moffitt Cancer Center & Research Institute, Tampa, Florida.; 4 Department of Anatomic Pathology, H. Lee Moffitt Cancer Center & Research Institute, Tampa, Florida.; 5 Department of Pharmaceutical Sciences, West Virginia University, Morgantown, West Virginia.; 6 BIO5 Institute, University of Arizona, Tucson, Arizona.; 7 Small Animal Imaging Laboratory, H. Lee Moffitt Cancer Center & Research Institute, Tampa, Florida.; 8 Department of Oncologic Sciences, University of South Florida, Tampa, Florida.; 9 Department of Physics, University of South Florida, Tampa, Florida.; 10 Department of Medical Engineering, University of South Florida, Tampa, Florida.

## Abstract

To increase the achievement of negative R_0_ surgical margins and increase the low survival rates of pancreatic cancer, improvements in assessing tumor margins during surgical resections are needed. This can be accomplished by using pancreatic cancer–targeted fluorescence molecular imaging agents to intraoperatively detect tumor margins in real time. Because Toll-like receptor 2 (TLR2) is broadly expressed among many cancer types including pancreatic adenocarcinomas, a high-affinity TLR2-targeted fluorescence molecular imaging agent (TLR2L-800) was developed. We investigate the potential for increased survival by employing real-time intraoperative tumor detection in a preclinical orthotopic human pancreatic xenograft tumor model using TLR2L-800. Three cohorts of nude mice bearing orthotopic human pancreatic xenograft tumors were intravenously injected with TLR2L-800. At 24 hours postinjection, one cohort underwent *in vivo* fluorescence-guided surgical removal of tumors using a real-time fluorescence imaging platform, a second cohort underwent visible light surgery (VLS), and a third cohort did not undergo surgery. A fourth, nontumor-bearing cohort was administered TLR2L-800 with no surgery. At 41 days postsurgery, the survival rates were 53% for the fluorescence-guided surgery (FGS) group and 0% for both the VLS and the tumor-bearing no-surgery group. The overall 200-day survival rate of 35% for the FGS group was significant compared with 0% for the VLS group (*P* value = 0.0018). This study demonstrates the potential of increasing disease-free survival for patients with pancreatic cancer by increasing the attainment of R_0_ margins using a novel tumor-targeted lipopeptide ligand-based fluorescence molecular imaging agent, TLR2L-800, during real-time FGS.

**Significance:**

Human TLR2 is broadly expressed among pancreatic adenocarcinomas, and the highly specific TLR2L-800 fluorescence molecular imaging agent has potential for use in fluorescence-guided surgery to increase R0 margins and improve patient survival.

## Introduction

Pancreatic ductal adenocarcinoma represents 90% of all pancreatic cancers (1). It is one of the most aggressive forms of cancer with an overall 5-year survival rate of 11% (2). Despite advances in treatment, surgical resection remains the best chance for a cure and long-term survival of pancreatic cancer. One of the important factors in increasing survival through surgery is the attainment of tumor-free surgical margins. Numerous reports have shown that patients with pancreatic cancer with negative microscopic margins (R_0_) have significantly improved survival compared with those with microscopic (R_1_) or macroscopic (R_2_) positive margins (3–5). In studies involving patients who survived long term, R_0_ negative resection margins were reported in 77% to 91% of patients who survived for more than 10 years (1, 6). Unfortunately, attainment of R_0_ margins following surgical resection of primary tumors is challenging and has been reported to range from 14% to 76% (7–9). This variability in achievement of negative margins is likely due to the lack of an international definition for resection margins and standardized protocols for pathologic examination. Even though the definition of margin clearance is still under debate, most studies now use a margin clearance over 1 mm to define a negative R_0_ resection. In comparing four large studies using a standardized protocol for margin reporting, the tumor-free margins were achieved in only 15% to 26% of surgical resections (10). Ultimately, positive resection margins, either microscopic or macroscopic, result in low survival, with rates comparable to patients who do not undergo surgery (11).

Typically, pancreatic cancer surgery is only performed for early-stage disease when complete resection of the tumor with negative margins is deemed possible. Resectability criteria are defined by the absence of distant metastases, absence of local tumor extension to the celiac axis and hepatic artery, and no involvement of the superior mesenteric vasculature. Unfortunately, only about 20% of patients are eligible to undergo surgery based on these criteria (12). To be eligible for a potentially curative surgical resection, a patient must first undergo extensive preoperative imaging and surgical planning. The conventional anatomic imaging modalities for preoperative imaging include multiphase intravenous contrast-directed thin-slice CT, MRI, endoscopic ultrasonography, and endoscopic retrograde cholangiopancreatography. There are limitations to using conventional anatomic imaging modalities for staging and surgical planning because translation to the surgical field is difficult, which lessens the number of eligible patients for resection (13). In particular, the limitations of preoperative imaging hinder the eligibility of patients with borderline resectable pancreatic cancer. Borderline resectable pancreatic cancer is neither clearly resectable nor clearly unresectable but more likely to yield an incomplete resection (14). Studies have found that one-third of unresectable pancreatic cancers were wrongly diagnosed due to limitations of preoperative imaging; these patients missed out on the opportunity to undergo curative resections (15). If better tools were available, the number of patients eligible to undergo pancreatic cancer surgery could be higher; thus, survival rates could also be higher. Alternately, the use of TLR2L-800 for diagnostic laparoscopy could reveal metastases not detected by preoperative imaging, potentially decreasing the number of unnecessary surgical procedures.

Besides using anatomic imaging modalities, the tools currently available to identify tumor from normal tissue during surgery are mostly limited to visual inspection and palpation. To modernize surgical methods, there is now significant interest in developing fluorescent molecular imaging agents and real-time fluorescence imaging systems for use in image-guided surgical navigation. Pancreatic cancer–targeted molecular imaging agents have been developed, and preclinical (16–18) and clinical (19–22) studies were performed. Development of new intraoperative surgical methods employing fluorescence-guided tumor detection could lead to increased negative resection margins (R_0_), resulting in improved survival rates of pancreatic cancer (23). The discovery of a target marker that is overexpressed on the surface of pancreatic cancer cells is the first step in the development of a targeted imaging agent with fluorescent label for use in intraoperative detection. We and others have reported that Toll-like receptor 2 (TLR2) is broadly expressed among many cancer types and has particularly broad and high expression among pancreatic adenocarcinomas (24, 25). TLR2, a member of the TLR family, is a type I transmembrane glycoprotein characterized by an external antigen recognition domain comprised of a highly conserved leucine-rich repeat motif, a transmembrane domain, and a cytoplasmic Toll/IL1 receptor homology signaling domain. Because TLR2 is a *bona fide* cell surface marker for pancreatic cancer that is highly expressed in 70% of pancreatic tumors but is not highly expressed in surrounding normal pancreas tissue or other surrounding tissues, fluorescence imaging probes developed using TLR2 ligands could be applied to the intraoperative detection of pancreatic tumor margins (26). Previously, a high-affinity TLR2-binding lipopeptide ligand conjugated to the Licor IRDye800CW near-infrared fluorescent dye, TLR2L-800, was synthesized and shown to have selectivity for TLR2-expressing tumors by *in vivo* fluorescence molecular imaging of pancreatic xenograft tumors in nude mice (26). TLR2L-800 has high TLR2-binding affinity (11 nmol/L K_i_) and high agonist activity (34 nmol/L EC_50_), and unconjugated TLR2L alone was shown to have potential for use as an immune adjuvant for cancer immunotherapy (26). In the current study, we performed additional *in vitro* and *in vivo* characterizations of TLR2L-800 and performed a preclinical fluorescence-guided surgery (FGS) study using TLR2L-800, mice bearing orthotopic human pancreatic cancer xenograft tumors and the SurgVision intraoperative open air fluorescence imaging T3 platform. The T3 platform is a surgical system that performs real-time fluorescence and white light imaging to produce an overlay of color and fluorescence images at video frame rates. Herein, improved rates of complete tumor resection and survival are demonstrated by FGS relative to controls.

## Materials and Methods

### Determining the absorbance and emission properties of TLR2L-800

Absorbance and fluorescence emission spectra were generated using a Tecan Infinite M-1000 PRO multimode microplate reader. Solutions of TLR2L-800 were prepared from a 0.367 mmol/L stock solution in DMSO. Aliquots (2 µL) of TLR2L-800 were spotted on each well of a Tecan NanoQuant plate. The absorbance spectrum of TLR2L-800 (183.5 µmol/L in PBS) was acquired from 230 to 1,000 nm with a wavelength step size of 2 nm. The emission spectrum of TLR2L-800 (0.367 mmol/L in DMSO) was acquired with an excitation wavelength starting from 275 nm and emission wavelengths from 450 to 850 nm with an emission wavelength step size of 2 nm and bandwidth of 5 nm.

### Determining the logD at pH 7.4 of TLR2L-800

The logD at pH 7.4 was determined by measuring the partition of TLR2L-800 in n-octanol and 25 mmol/L NaH_2_PO_4_/Na_2_HPO_4_ aqueous buffer pH 7.4. Briefly, TLR2L-800 was mixed with octanol and the phosphate buffer via vortexing, and the fluorescence signal of each layer was measured using the IVIS200 imaging system (PerkinElmer), with setting excitation (710–760 nm) and emission (810–875 nm). The logD7.4 was calculated based on the fluorescence signals ratio between octanol layer and phosphate buffer layer.

### *In vitro* profiling of TLRs

TLR agonist activity profiling was performed by InvivoGen. For these assays, 1 μg/mL of test compounds and known control agonists for each TLR were tested in triplicate for induction of the secreted embryonic alkaline phosphatase (SEAP) reporter by the transcription factor NF-κB in HEK293 cells; each cell line was engineered to express one of the human TLRs and SEAP. In a 96-well plate (200 μL total volume) containing the appropriate cells (50,000–75,000 cells/well), 20 μL of the test compound or the positive control ligand is added to the wells for a final concentration of 1 μg/mL. The media added to the wells were designed for the detection of NF-κB–induced SEAP expression. After 16 to 24 hours of incubation, the optical density was read at 650 nm on a Molecular Devices SpectraMax 340PC absorbance detector. To further demonstrate selectivity to TLR2, additional *in vitro* TLR2-negative control agonist screenings were performed using non–TLR2-expressing control cell lines, HEK293/Null 1, HEK293/Null k, and HEK293/Null2. TNFα was used as the positive control as it activates NF-κB signaling independent of the TLR2. For each test agent and control, three independent assays were performed in triplicate.

### Cell culture

For the mouse model, SU.86.86 human pancreatic adenocarcinoma cells (ATCC CRL-1837) were grown in RPMI 1640 media (Life Technologies, Gibco) supplemented with 10% FBS (VWR Seradigm Radnor). We have previously reported that SU.86.86 cells endogenously overexpress TLR2 on the cell surface (26, 27). Cells were grown at 37°C and 5% CO_2_. Prior to and upon completion of experiments, cell lines were authenticated using short tandem repeat DNA typing according to ATCC’s guidelines (28).

### Animals

All procedures were performed in compliance with the Guide for the Care and Use of Laboratory Animal Resources (1996), National Research Council, and approved by the Institutional Animal Care and Use Committee, University of South Florida, under the approved protocol numbers, R4002 and R0946. Immunocompromised mice were housed in a clean facility with special conditions that include HEPA-filtered ventilated cage systems, autoclaved bedding, autoclaved housing, autoclaved water, irradiated food, and special cage changing procedures. Mice were handled under aseptic conditions including the wearing of gloves, gowns, and shoe coverings. Mice were anesthetized by inhaled isofluorane gas (flow 2–2.5 L/minutes) and remained anesthetized for the minimum amount of time required for imaging and surgical studies, ranging from 3 to 45 minutes at a time. Four days prior to fluorescence imaging, the mice were switched to fluorescence imaging feed (Harlan AIN 93G) to help minimize internal background fluorescence. Health checks and weighing of mice were performed daily. Euthanasia occurred at the experimental endpoint of 200 days, when mice lost more than 20% body weight rapidly or progressively, appeared emaciated, had ulcerated/necrotic tumors, or other observations of protocol specified clinical end points. Only female mice were used because, in our experience, male mice have a greater incidence of fighting and damaging tumors or wounding cage mates.

### Subcutaneous human pancreatic cancer xenograft mouse model

Human SU.86.86 pancreatic cancer cells (8 × 10^6^) were xenografted into the right flanks of female athymic nude mice 6 to 8 weeks old (Harlan). Weights and tumor volume measurements were performed biweekly. Tumor volumes were determined using caliper measurements and the formula: volume (mm^3^) = (length × width^2^)/2. After 4 weeks, imaging studies were performed once the tumors reached an average size of 500 mm^3^.

### Pharmacokinetics and biodistribution

To evaluate the pharmacokinetics and biodistribution of TLR2L-800 after administration of a single, 100 nmol/kg dose in a volume of 100 μL, both *in vivo* and *ex vivo* fluorescence images were acquired using the Xenogen IVIS 200 Series Imaging System (PerkinElmer Caliper Life Sciences). Excitation (710–760 nm) and emission (810–875 nm) filters were used in wavelength ranges suitable for *in vivo* excitation and detection of emitted light of IRDye800CW. Acquisition times ranged from 4 to 10 seconds to keep intensity counts above a minimum of 15,000 but below saturation values of ∼60,000. Results are displayed in the Living Image 4.2 software (PerkinElmer Xenogen Caliper Life Sciences) recommended radiant efficiency units [(p/seconds/cm^2^/sr)/(µW/cm^2^)], in which the epi-fluorescence values (fluorescence emission radiance per incident excitation power) within an image are normalized using a stored reference image that represents the variation in excitation light intensities across the stage. This unit of measure allows for a more quantitative comparison of fluorescence from images acquired at different times and locations on the stage. Images were acquired prior to injection to establish a baseline and immediately after injection to check for successful injection. The data were analyzed using Living Image 4.2 software after performance of instrument background subtraction and tissue autofluorescence subtraction using a preinjection image and identical regions of interest (ROI). ROIs were drawn, and mean radiant efficiency was calculated. Uptake and clearance parameter estimations were performed using nonlinear regression analysis tools available in GraphPad Prism v6.02 as described in the “Statistical information” section.

### Orthotopic human pancreatic cancer xenograft mouse model

Female athymic nude mice (Harlan) 6 to 8 weeks old underwent ultrasound guided injection of 1 × 10^6^ human pancreatic cancer cells (SU.86.86) directly into the pancreas to create orthotopic pancreatic cancer xenograft tumors according to methods previously described (29). Mice were weighed biweekly. Tumor growth was monitored weekly by tomographic ultrasound imaging.

### *In vivo* pancreatic tumor surgical resections

Mice underwent surgery to remove pancreatic tumors in a cohort survival study as outlined in Supplementary Fig. S1. Female nude mice bearing orthotopic SU.86.86 human pancreatic cancer xenograft tumors were generated using the methods described in the “Orthotopic human pancreatic cancer xenograft mouse model” section. All mice received 100 nmol/kg TLR2L-800 in a 100 µL volume injected into the tail vein 24 hours prior to surgery. The surgeries were performed by using aseptic techniques and rodent survival surgical procedures. Mouse preparations were performed in a location remote from the operating area designated as the pre-op preparatory area. Mice received buprenorphine at 0.1 to 2.0 mg/kg subcutaneously and ketoprofen at 10 mg/kg subcutaneously, preemptively for pain management. Mice were anesthetized and positioned in right lateral recumbency. The area just below the costal border of the thorax was prepared with three circular (inside to outside) scrubs utilizing a surgical scrub; alcohol wipes were performed between each scrub, and a final betadine paint was applied over the area. One mouse at a time was then transferred to the sterile surgical field. A skin incision approximately 1.0 to 1.5 cm was made below the last rib. The pancreas was manipulated and positioned to allow visualization of and access to the tumor. The surgeon manually resected all tumor tissues observed. The remaining pancreas was placed back within the abdominal cavity; the muscle wall was brought into apposition and held with absorbable sutures (4-0 Vicryl), and the skin layer closed with wound clips. Next, the mouse was taken to the post-op area for recovery. Mice received buprenorphine at 0.1 to 2.0 mg/kg subcutaneously and ketoprofen at 10 mg/kg subcutaneously every 12 hours for 48 hours post-op and then provided as needed for the next 48 hours. Clips were removed at 10 to 14 days post-op. Mice were observed daily for the entire duration of the study. Euthanasia occurred at either the experimental endpoint of 200 days or when mice exhibited signs of protocol specified clinical end points as previously described in the “Animals” section. The entire aseptic surgical procedure was repeated for each individual mouse.

Prior to surgery, mice were randomly split into four groups to study survival rates: FGS (*n* = 17), visible light surgery (VLS; *n* = 13), tumor-bearing no-surgery mice (*n* = 13), and nontumor-bearing no-surgery mice (*n* = 3). For the FGS group, *in vivo* fluorescence-guided surgical removal of pancreatic tumors was performed by resecting tumors using real-time fluorescence imaging. Prepared mice were transferred to the fluorescence imaging system, and the pancreatic tumors were identified by TLR2L-800–labeled fluorescence as observed on the imaging station monitor. This survival surgery study was performed in three different rounds occurring at different time intervals. The first round of FGS was performed using an adapted version of the LightTools system outfitted with an ICG compatible filter set (748–789-nm excitation wavelength; 814–851-nm emission wavelength), a cooled CCD camera, and SPOT Imaging Advanced software V4.7 (Diagnostic Instruments). For this system, the mice are placed inside a dark chamber for the surgical resections which required the surgeon to observe via the monitor or through a small observation port at the top of the dark chamber (Supplementary Fig. S2A). After obtaining successful results using this system, the two subsequent rounds were performed using an intraoperative open air fluorescence imaging instrument, T3 Imaging Platform equipped with a 750-nm laser (SurgVision; Supplementary Fig. S2B). For the VLS group, surgeries were performed under standard conditions with visible light guiding the surgeon’s ability to identify tumors. Once mice were prepared for surgery, one mouse at a time was transferred to a standard operating table. The tumor-bearing no-surgery mice and nontumor-bearing no-surgery mice did not undergo surgical resections.

### Ultrasound imaging

The Vevo 2100 Imaging Station (FUJIFILM VisualSonics) was used for all ultrasound imaging. Image acquisitions were performed using the enhanced abdominal measurement package in the B-mode and 3-D mode settings. Physiologic status (electrocardiogram, respiration, blood pressure, and body temperature) of the mice was closely monitored during each image acquisition session. Mice were imaged prior to tumor xenografting to establish baseline images and then imaged weekly for up to 6 weeks prior to surgery using ultrasound to monitor growth of the orthotopic pancreatic tumors. Mice were also imaged weekly postsurgery to monitor tumor recurrence.

### Fluorescence molecular tomographic imaging

*In vivo* fluorescence molecular tomography (FMT) imaging using the FMT2500 (PerkinElmer) was also performed pre- and postsurgery to confirm presence of tumor and assess if the surgeries resulted in complete resection of the orthotopic tumor. TrueQuant v3.1 software (PerkinElmer) was used to draw ROIs over the tumors to determine the mean TLR2L-800–related fluorescence in the region of the pancreas *in vivo*.

### Histologic analysis

Histologic analyses were performed on the surgically resected tumors and pancreas tissues. Samples were fixed in 10% formalin solution, processed, embedded, sectioned, and stained with hematoxylin and eosin (H&E) for the presence of tumor or with TLR2-specific antibody (GeneTex, # GTX31279) for IHC determination of target expression.

### Statistical information

Data are represented as mean ± SD. All statistical analyses were performed with GraphPad Prism v7.03. The unpaired Welch’s *t* test was used to determine the statistical significance of differences between two independent groups of variables, in which *P* ≤ 0.05 was considered significant. A Gaussian distribution was assumed with the use of parametric tests, whereas the unpaired *t* test with Welch’s correction did not assume equal SDs. Clearance and decay parameters for the pharmacokinetics and biodistribution studies were statistically analyzed using nonlinear regression analysis. Both one-phase and two-phase exponential decay equation models were used to determine the best line fits for the data plots. Survival curve comparisons were calculated using a log rank test method, also referred to the Mantel–Cox method.

### Data availability

The data generated in this study are available upon request from the corresponding author.

## Results

### Absorbance and emission properties of TLR2L-800

As the absorbance and emission maxima of a fluorochrome can change following conjugation, it is important to determine these values for any novel conjugate to enable use of the optimal fluorescence imaging instrument settings. Absorbance and fluorescence emission spectra were acquired for TLR2L-800 (Supplementary Fig. S3). The absorbance and emission maxima of TLR2L-800 were determined to be 786- and 832-nm wavelengths, respectively. These values differ from the values reported by the LI-COR product sheet for unconjugated IRDye800CW in water, i.e., 774 and 789 nm, respectively.

### Lipophilicity of TLR2L-800

As lipophilicity can influence the tissue distribution and tumor uptake of an agent, the logD at pH 7.4 of TLRL2-800 was determined to be −2.90 ± 0.15 as described in the “Materials and Methods” section.

### *In vitro* human TLR ligand selectivity

**Figure 1:**
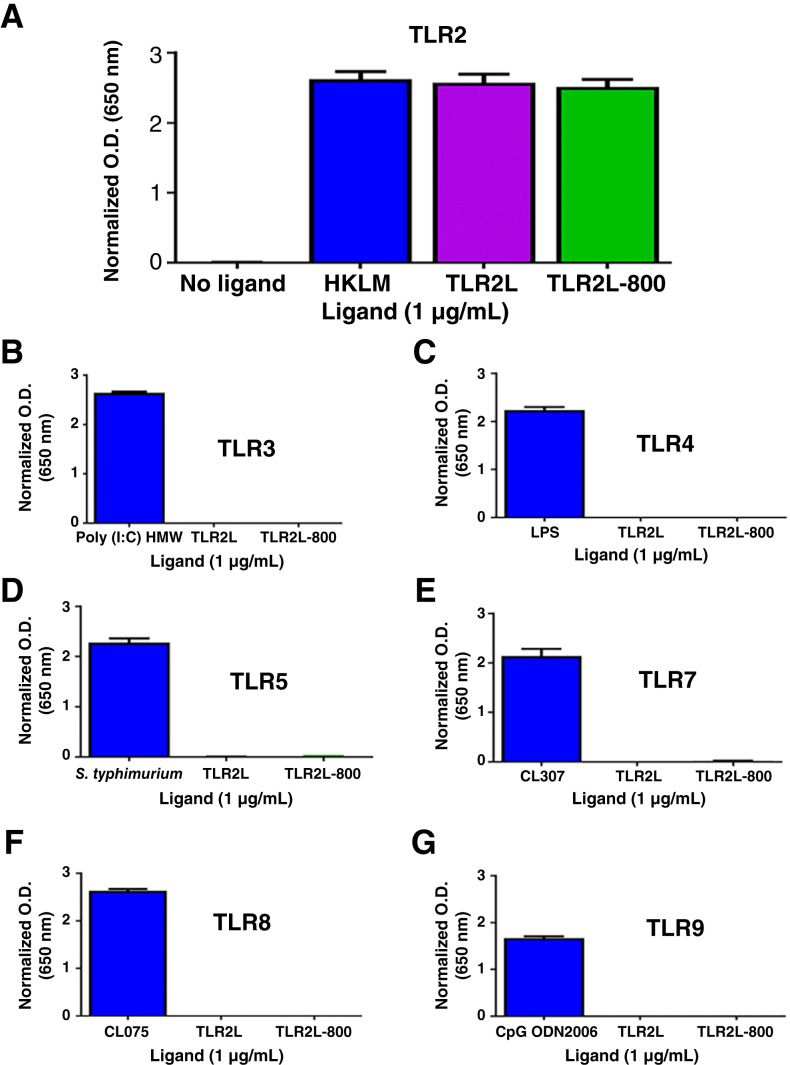
*In vitro* human TLR ligand selectivity. Unlabeled TLR2L (shown in purple) and the fluorescent-labeled version, TLR2L-800, (shown in green) underwent agonist activity screening for the human TLRs to determine selectivity: (**A**) TLR2, (**B**) TLR3, (**C**) TLR4, (**D**) TLR5, (**E**) TLR7, (**F**) TLR8, and (**G**) TLR9. Known agonist ligands for each TLR were used as positive controls (shown in blue; *n* = 3 assays per TLR).

TLR2L-800 and the unconjugated ligand, TLR2L, were screened for agonist activity on the full complement of human TLRs (2, 3, 4, 5, 7, 8, and 9). Figure 1A shows comparable TLR2 agonist activity for TLR2L, TLR2L-800, and the positive control, HKLM. Furthermore, TLR2L and TLR2L-800 had no agonist activity for the other human TLRs (Fig. 1B–G). To further demonstrate the specificity of TLR2L and TLR2L-800, TLR-negative cell lines were screened for agonist activity, and little to no activity was observed (Supplementary Fig. S4).

### Pharmacokinetics and biodistribution of TLR2L-800

**Figure 2:**
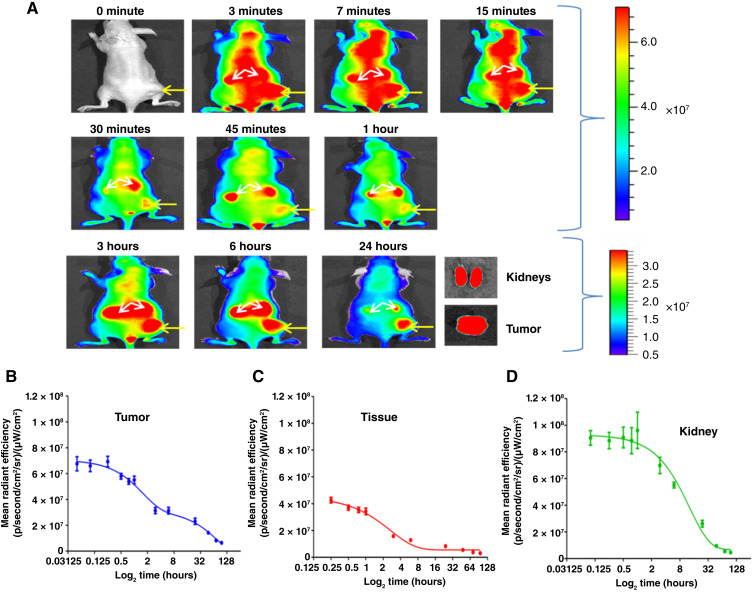
*In vivo* pharmacokinetics of 100 nmol/kg TLR2L-800 in mice bearing subcutaneous TLR2^+^ (SU.86.86) pancreatic tumor xenografts on the right flank. **A,** The uptake of TLR2L-800 was observed in the tumor (yellow arrow), kidneys (white arrows), and surrounding tissue in representative *in vivo* fluorescence images acquired from 0 to 24 hours. For confirmation of *in vivo* TLR2L-800 uptake, tumor and kidneys were also imaged *ex vivo* at 24 hours. Graphs show the *in vivo* clearance profiles for (**B**) tumor, which exhibited a two-phase exponential decay linefit, whereas clearance from the (**C**) tissue and (**D**) kidney was best fitted by a one-phase exponential decay equation (*n* = 9). Note: refer to Table 1 for statistical data of the *in vivo* clearance profile graphs.

To better understand the timing of when tumors can be first resolved from surrounding tissue and the timing of agent clearance from circulation, we performed an epi-fluorescence radiance efficiency image acquisition time course study using nude mice bearing subcutaneous SU.86.86 xenograft tumors, *n* = 9 (Fig. 2A). The rate of TLR2L-800 clearance was fitted for tumor, normal tissue, and kidney using exponential decay regression analyses (Fig. 2B–D). A two-phase exponential decay provided the highest goodness of fit for tumor clearance, with an initial fast clearance from 3 minutes to 3 hours and a slow clearance from 3 to 96 hours. The normal tissue and kidneys were best fit using a one-phase exponential decay regression (Table 1). As the timing of the fast tumor clearance and the normal tissue clearance is comparable (1.1- vs. 1.9-hour half-life, respectively), this likely corresponds to the timing of TLR2L-800 blood clearance. As the tumors express TLR2 and the normal tissue does not, the slow tumor clearance likely corresponds to receptor mediated tumor cell uptake (55 hours half-life). We were not able to acquire multiple images during the initial rapid uptake, so the uptake kinetics were not estimated using these data. However, we have previously estimated the uptake kinetics of TLR2L-800 using intravital fluorescence image acquisitions and a mathematical model (27). At 24 hours postinjection of the TLR2L-800 agent, *ex vivo* fluorescence images were acquired of major organs removed from an agent-injected tumor-bearing no-surgery mouse and a non-injected tumor-bearing no-surgery mouse (Supplementary Fig. S5A). The fluorescence signals from the control organs were subtracted from the fluorescence in the corresponding organs of the agent-injected mouse. These *ex vivo* data further confirm that TLR2L-800 agent uptake is primarily observed in the tumor and kidneys (Supplementary Fig. S5B). Supplementary Figure S5C shows TLR2 target expression in SU.86.86 xenograft tumors.

**Table 1:** Nonlinear regression analyses of clearance of 100 nmol/kg TLR2-800 in TLR2-negative tissues and TLR2-positive tumors

ROI	Fit	Time range, hour	Rate constant, *K* (SE)	Half-life, hour	*R* ^2^	*n*
Tumor	Two-phase exponential decay	0.05–96	*K*_fast_ = 0.65 (0.2)	Fast = 1.1	0.99	12
			*K*_slow_ = 0.013 (0.2)	Slow = 55		
Tissue	One-phase exponential decay	0.25–96	0.38 (0.06)	1.9	0.98	10
Kidneys	One-phase exponential decay	0.10–96	0.078 (0.01)	8.8	0.99	11

**Figure 3:**
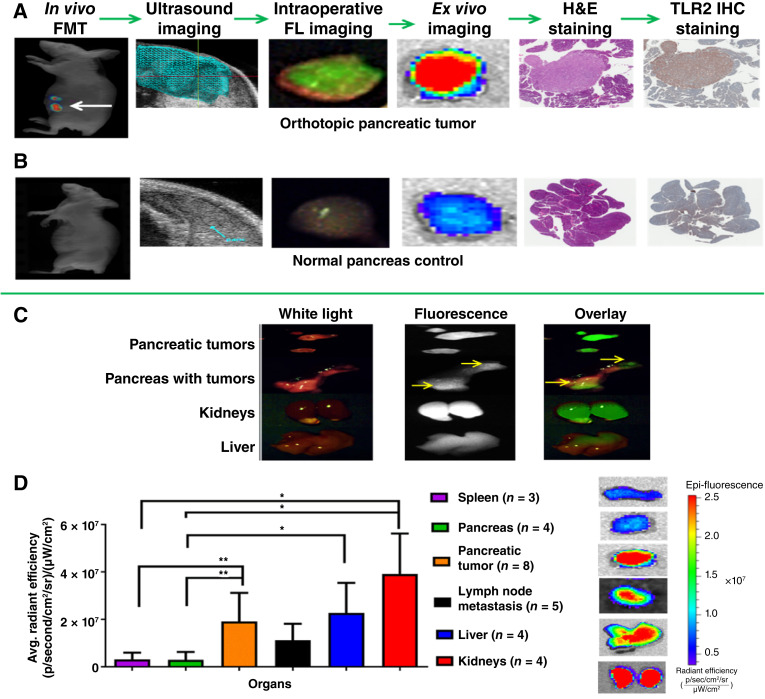
Pharmacokinetics and biodistribution of 100 nmol/kg TLR2L-800 in an orthotopic human pancreatic cancer xenograft mouse model. **A,** Representative images from one animal that include an *in vivo* presurgery FMT image showing fluorescence in the region of the pancreas (white arrow), an *in vivo* 3D ultrasound image of the tumor, *ex vivo* fluorescence overlay and reflectance images of the resected tumor using the SurgVision and IVIS imaging systems, H&E staining showing the presence of tumor surrounded by normal tissue, and IHC staining of TLR2 expression in the tumor. **B,** The corresponding image sequence of tumor-free pancreas tissue is shown for a nontumor-bearing no-surgery mouse. **C,** Representative *ex vivo* fluorescence images, acquired using the SurgVision MSNI open air platform, of resected pancreatic tumors, pancreas, kidneys, and liver 24 hours postinjection are shown (*n* = 4). **D,** Graph shows the mean quantified *ex vivo* fluorescence in tumors and organs at 24 hours postinjection (*n* = 4 mice). Asterisks indicate significance: *, *P* < 0.01; **, *P* < 0.05.

The *in vivo* pharmacokinetics and biodistribution of TLR2L-800 were also evaluated in the SU.86.86 orthotopic pancreatic xenograft tumor model (29). First, a tumor-bearing mouse was administered 100 nmol/kg TLR2L-800 and, at intervals from 0 to 168 hours postinjection, *in vivo* FMT images were acquired (Supplementary Fig. S6). Fluorescence above background was observed in the regions of the pancreas, kidneys, and liver throughout the timecourse; and by 24 hours, the background fluorescence had cleared from other surrounding normal tissues. The comparison of uptake of TLR2L-800 in representative orthotopic tumor-bearing mice administered TLR2L-800 versus representative nontumor-bearing no surgery that also received TLR2L-800 is shown in Fig. 3A and B using various imaging modalities. Figure 3A shows representative presurgery FMT and ultrasound images of a tumor-bearing mouse, and corresponding fluorescent tumor during surgery, as well as corresponding *ex vivo* fluorescence, H&E staining, and TLR2 IHC staining images (*n* = 4). Staining confirmed the presence of tumor within surrounding normal pancreas tissue and TLR2 expression in the tumors but not in surrounding pancreas tissue. For comparison and to validate the selectivity of TLR2L-800, nontumor-bearing mice underwent nonsurvival fluorescence-guided resection of the normal pancreas 24 hours postinjection. TLR2L-800–associated fluorescence was not observed in the pancreata, and staining confirmed that there were no tumors and that the pancreata were negative for TLR2 expression (Fig. 3B). Next, *ex vivo* fluorescence images were acquired using the SurgVision MSNI open air platform of resected pancreatic tumors, pancreas, kidneys, and liver 24 hours postinjection, *n* = 4 mice (Fig. 3C). To quantify tissue distribution at 24 hours postinjection, tissues were rendered, and fluorescence in the acquired *ex vivo* fluorescence images was quantified per organ, *n* = 4 mice (Fig. 3D). Significantly elevated fluorescence was observed in pancreatic tumor relative to normal pancreas (*P* < 0.01), as well as significantly elevated fluorescence in the liver and kidney relative to normal pancreas and spleen (*P* < 0.05; *n* = 4). Fluorescence was also detected above background for lymph node metastases (*n* = 5 metastases from four mice).

### Tumor-free survival with TLR2-targeted FGS

**Figure 4:**
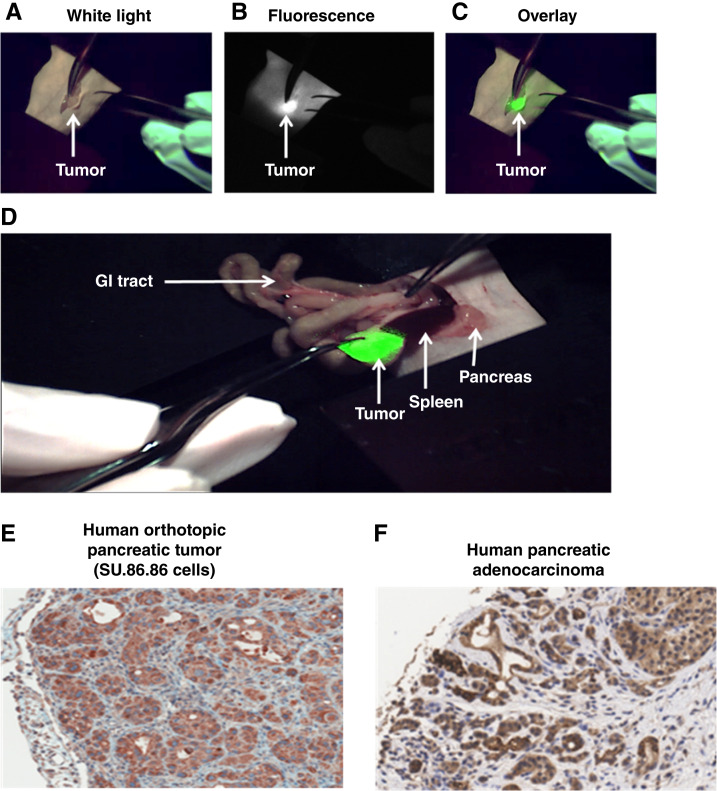
Representative images showing the real-time FGS resection of the fluorescently labeled orthotopic pancreatic tumor (green) 24 hours postinjection of TLR2L-800 from the survival surgery study. Images were acquired using the SurgVision MSNI open air imaging platform showing (**A**) visible light, (**B**) fluorescence, and (**C**) the overlay of green fluorescence on the corresponding visible light image. **D,** The intraoperative detection of the fluorescently labeled orthotopic pancreatic tumor (green) was easily identified from normal pancreas tissue and other organs, such as spleen and gastrointestinal tract. TLR2 immunohistostaining and expression patterns in (**E**) the SU.86.86 orthotopic pancreatic tumor model in mice compared (**F**) a human pancreatic tumor tissue sample, in which the stromal component and TLR2 IHC staining are comparable in both samples.

**Figure 5:**
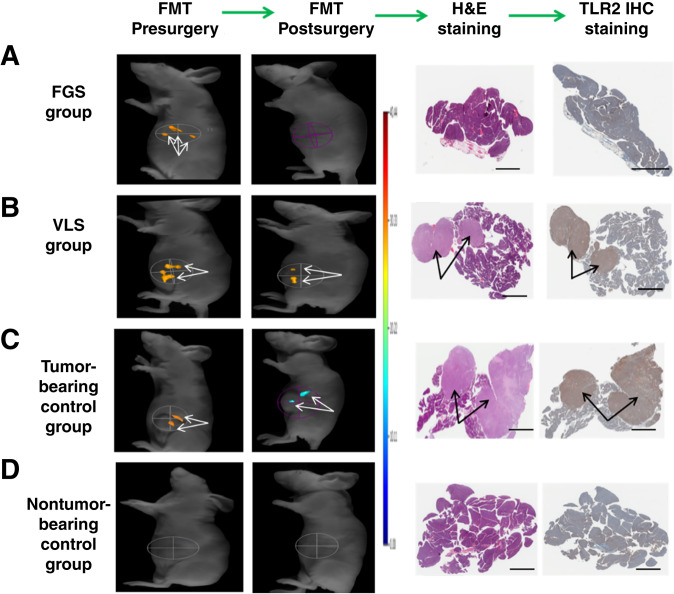
Identification of tumor presence in each group of the survival surgery study. For (**A**–**D**), shown are representative pre- and postsurgery *in vivo* FMT images with corresponding H&E and TLR2 IHC staining of each group in the survival surgery study, indicating the presence or absence of orthotopic pancreatic tumors.

To investigate the potential of achieving tumor-free R_0_ margins and improving survival rates using TLR2L-800, *in vivo* pancreatic tumor resections were performed using human SU.86.86 orthotopic pancreatic xenograft tumors in nude mice according to the study protocol flow chart (Supplementary Fig. S1). All mice received an i.v. injection of 100 nmol/kg TLR2L-800; at 24 hours postinjection, *in vivo* ultrasound and FMT images were acquired to verify the presence of tumors. The 24-hour timepoint was selected based on the observation that optimal tumor uptake and tissue clearance occur between 6 and 24 hours (Fig. 2B and C). Mouse cohorts were randomly split into four different groups: FGS (*n* = 17), VLS (*n* = 13), tumor-bearing no-surgery (*n* = 13), and nontumor-bearing no-surgery (*n* = 3). Mice in the FGS group were prepared for surgery and transferred to the sterile surgical field (Supplementary Fig. S2A and S2B). By viewing the fluorescence imaging system monitor in real time, the surgeon located the fluorescently labeled pancreatic tumor *in situ* after making the initial surgical incision (Fig. 4A–D). The intraoperative detection of the fluorescently labeled orthotopic pancreatic tumors was easily identified from normal pancreas tissue and other organs, and these fluorescently labeled tumors were resected. The VLS group of mice underwent a similar surgical resection using normal visible light conditions 24 hours postinjection of TLR2L-800. *Ex vivo* fluorescence imaging and histologic analyses performed on the resected pancreatic tumors determined that all resected tumors were fluorescently labeled and TLR2-positive. It is notable that the human SU.86.86 orthotopic pancreatic cancer xenograft tumors exhibited a stromal component similar to that observed in pancreatic tumor tissue from human patients. TLR2 IHC and histologic counterstaining in a section of pancreatic cancer tumor from a human patient and in a section of an SU.86.86 orthotopic xenograft have comparable patterns of TLR2-expressing tumor cells (brown stain) and infiltrated stroma (blue stain; Fig. 4E and F). For all mice in the survival study, pre- and postsurgery FMT images and *ex vivo* images of the resected tissue were acquired, and histologic staining was performed (Supplementary Table S1). In every case, tumor-specific TLR2L-800 fluorescence was observed in tumor-bearing animals presurgery, and no TLR2L-800–related fluorescence was observed in nontumor-bearing no-surgery controls (Fig. 5). In most cases, there was no fluorescence observed in the region of the pancreas post-FGS (Fig. 5A), whereas in nearly every case, fluorescence remained following VLS (Fig. 5B). For comparison, tumor-bearing and nontumor-bearing no-surgery control mice that did not undergo surgery were also imaged and shown in Fig. 5C and D. Some (24%) of the mice in the FGS cohort had complete pancreatic tumor resections, but these mice had TLR2L-800 fluorescence-labeled metastases in other regions that were identified by FMT: liver (*n* = 1), bladder (*n* = 1), abdominal (*n* = 3), and axillary lymph node (*n* = 1; Fig. Supplementary S7; Supplementary Table S2). Incomplete resections were observed in 41% of the FGS group (*n* = 7) and 100% of the VLS group (*n* = 13; Table 2).

**Table 2:** Outcomes for the different types of pancreatic cancer surgical resections

Cohort	Surgical resection type	*n* value (%)	Mean progression to endpoint (days)
FGS	Overall	17 (100)	90
	Complete pancreatic tumor resection, no metastases present	6 (35)	200
	Complete pancreatic tumor resection, metastases present	4 (24)	19
	Incomplete pancreatic tumor resection	7 (41)	37
VLS	Incomplete pancreatic tumor resection	13 (100)	27
Tumor-bearing no surgery	No pancreatic tumor resection	13 (100)	25
Nontumor-bearing no surgery	No resection	3 (100%)	200

**Figure 6:**
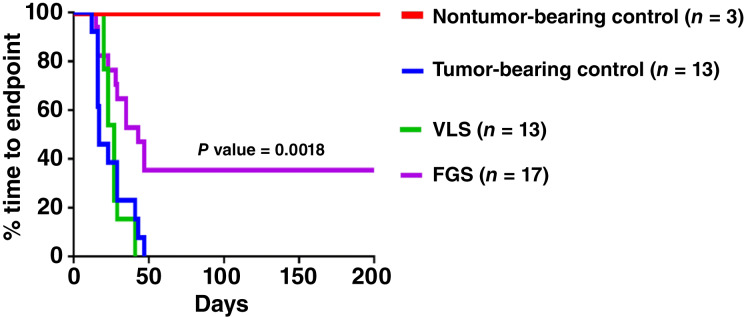
Kaplan–Meier analysis of the time to endpoint for animals in each grouping. The overall 35% survival rate for the FGS group is significantly higher than that of the VLS group (*P* value = 0.0018).

Because this was a survival surgery study, mice were observed daily until a clinical endpoint was observed, or the experimental endpoint of 200 days postsurgery was reached. The experimental endpoint coincided with the typical life span of ∼1 year and animals that reached this endpoint were typically exhibiting signs of old age. After 41 days postsurgery, 53% of the animals in the FGS group were still alive, whereas 0% of the VLS group and only 15% of the tumor-bearing no-surgery group were still alive (Fig. 6; Supplementary Table S2). By 47 days postsurgery, 35% of the FGS group remained alive, and these animals continued to live tumor-free until the experimental endpoint. The mean progression to endpoint for the FGS, VLS, tumor-bearing no-surgery, and nontumor-bearing no-surgery groups were 90, 27, 25, and 200 days, respectively (Table 2). The mean progression to endpoint for the FGS group was significantly longer than that of the VLS group (*P* value = 0.002). Mice in the FGS group that had incomplete resections (41%) had an increased mean progression to endpoint (12 days) compared with the tumor-bearing no-surgery controls. The VLS group had only a 2-day increase in progression to endpoint versus the tumor-bearing no-surgery controls.

After the clinical or experimental end point was reached for each animal, the pancreas and other organs were resected *postmortem* to undergo histologic and pathologic evaluations for presence of tumors or metastases (Table 2). Of the FGS mice that survived to the 200-day experimental endpoint (35%), pathology determined that all were cancer free. Of the FGS mice that reached clinical endpoint prior to 200 days, 24% had no pancreatic tumors but did have metastases and 41% had pancreatic tumors. Hence, 59% of the FGS procedures resulted in complete resection. However, 100% of the VLS group had incomplete resections and reached clinical endpoints prior to 200 days with a mean progression to endpoint of 27 days (Table 2).

## Discussion

Herein, we report both *in vitro* and *in vivo* preclinical characterizations of our previously reported TLR2L-800 fluorescence molecular imaging probe (26), and its potential for use in the FGS of pancreatic cancer. We have previously reported the high and broad expression of TLR2 among human pancreatic tumors and low expression of TLR2 in most non-vital tissues (30). Because high TLR2 expression has also been reported in other cancers and diseases (30), as well as our observations of fluorescently labeled TLR2-expressing metastases in this study, TLR2L-800 may potentially be used to fluorescently detect a broad range of pathologies.

The chemical, optical, and biological properties of TLR2L-800 were further characterized in this study. Conjugation of the IRDye800CW to the TLR2L targeting ligand generated a 12-nm shift in the excitation maxima and a 43-nm shift in the emission maxima to longer wavelengths relative to unconjugated dye, resulting in an increase from a 15- to a 46-nm Stokes shift. However, this shift does not impair the detection of the TLR2L-800’s fluorescence signal using near-infrared fluorescence imaging systems. The lipophilicity of TLR2L-800 was also determined, verifying the solubility of the conjugated agent. Due to the conserved sequence homology amongst the TLR family, it is possible for TLR targeted ligands to exhibit specificity toward multiple family members. For example, E567 is a small molecule inhibitor of both TLR2 and TLR4 (31). However, *in vitro* bioassays confirmed the specificity of TLR2L-800 for the TLR2 member of the TLR family. TLR1 and TLR6 were not included in these assays as individual targets because they only function as signaling receptors when dimerized with TLR2 (32). However, the cell lines used for the screening assays were derived from HEK293 cells which express endogenous levels of both TLR1 and TLR6. We had previously reported the specificity of the TLR2L and TLR2L-800 ligands for TLR2/TLR6 or TLR2/TLR1 agonism (26).

As reported for other fluorescence molecular imaging agents, (33, 34) the pharmacokinetics and biodistribution of the TLR2L-800 agent were evaluated using mice bearing subcutaneous and orthotopic SU.86.86 human pancreatic xenograft tumors and agent-related fluorescence as a surrogate for concentration. By administering a range of concentrations, we previously established that a 100 nmol/kg dose of TLR2L-800 provided sufficient tumor to surrounding tissue contrast for fluorescence imaging at the 24 hours postinjection timepoint (26). Although tumor-specific uptake of TLR2L-800 had been previously established for TLR2L-800 in orthotopic human pancreatic xenograft tumors with endogenous expression of TLR2 (26), pharmacokinetics and biodistribution studies herein determined: low uptake of TLR2L-800 in normal pancreas tissue, high uptake of TLR2L-800 in orthotopic human pancreatic xenograft tumors with endogenous expression of TLR2, and clearance of TLR2L-800 via the renal and hepatic pathways. Clearance kinetics were determined for tumors, kidneys, and normal tissues. Clearance from normal pancreas tissue was shown to occur within 24 hours postadministration.

To improve the intraoperative detection of surgical margins leading to increased survival rates of pancreatic cancer, targeted FGS were performed on preclinical orthotopic human pancreatic cancer xenograft tumor models. The fluorescence molecular imaging agent, TLR2L-800, was used to intraoperatively detect tumors using a real-time fluorescence imaging system. Animals that underwent FGS relative to VLS had a significantly longer progression to endpoint (*P* value 0.002). A large percentage (35%) of FGS mice lived-out the preponderance of their natural lifespan tumor-free, until the 200-day postsurgery experimental endpoint. In comparison, none of the VLS animals reached the experimental endpoint, and all had pancreatic tumors upon reaching the clinical endpoint. Because 24% of the FGS mice that reached clinical endpoints had complete primary tumor resections but succumbed to metastasis burden, a complete resection rate of 59% was observed in this study. Mice in the FGS group that had incomplete resections (41%) had an increased mean time to clinical endpoint (12 days) compared with the tumor-bearing animals that did not undergo surgery. However, the incomplete resections performed on the VLS group only extended survival by an average of 2 days longer than the tumor-bearing no-surgery mice. The FGS cohorts that survived past 47 days did not exhibit any signs of tumor recurrence. Overall, these results suggest that mice left with positive margins had low survival rates, similar to mice that did not undergo surgery; whereas complete resections resulted in increased survival or complete cure. Other preclinical studies have also reported increased pancreatic cancer survival by FGS (16, 35, 36).

The goal of this study was to generate preclinical data that will support the translation of TLR2L-800 for use in FGS for pancreatic cancer. To this end, we used a human orthotopic pancreatic cancer xenograft model that has a stromal component similar to human disease. TLR2L-800 was shown to penetrate throughout these tumors via *ex vivo* fluorescence imaging of tumor sections. For our survival surgery study, the intraoperative SurgVision system was used. This platform is currently being used in clinical trials that involve FGS for various cancers, such as pancreatic, breast, colorectal, esophageal, ovarian, and glioblastoma (37–42). However, these studies for intraoperative detection of pancreatic cancer are using antibodies labeled with the IR800 dye; our approach utilizes a novel tumor-targeted lipopeptide ligand-based fluorescence imaging agent. Small molecules, like the TLR2L ligand, are believed to exert better tumor penetration, less non-specific binding and are typically cleared faster from the circulation than larger antibody-based agents, resulting in better tumor-to-background ratios (43).

We have demonstrated that the intraoperative detection of fluorescently labeled pancreatic tumors targeted by TLR2L-800 provides surgical guidance that potentially aids in the identification of tumor margins that are not readily distinguishable from adjacent normal tissue under normal white light conditions. In our surgical studies, when no fluorescence was observed in the pancreas following surgery, we assumed that the surgery provided clean margins. We anticipate that surgical resections of pancreatic cancer guided by the intraoperative detection of tumor-targeted fluorescence will enhance the real-time assessment of tumor margins, thereby improving the rates of achieving R_0_ margins. This may increase the number of patients eligible for surgical resections, leading to better outcomes and higher survival rates.

## Supplementary Material

Figure S1 Flow chart for the tumor-free survival with TLR2 targeted fluorescence-guided surgery study.

Figure S2 In vivo fluorescence-guided surgical imaging systems.

Figure S3 Optical characterization of TLR2L-800.

Figure S5 Ex vivo biodistribution of 100 nmol/kg TLR2L-800 in mice bearing subcutaneous TLR2+ (SU.86.86) pancreatic tumor xenografts on the right flank.

Figure S6 Pharmacokinetics and biodistribution of 100 nmol/kg TLR2L-800 in an SU.86.86 orthotopic human pancreatic cancer xenograft mouse model.

Figure S7 In vivo fluorescence molecular tomographic images of TLR2L-800 labeled SU.86.86 orthotopic pancreatic tumors in nude mice 24 h post-surgery.

Figure S4 In vitro TLR2-negative agonist activity control.

Table S1 Expression of pancreatic tumors identified by various methods for the 4 survival study cohorts.

Table S2 Identification of pancreatic tumors and suspected cause of death for individual mice.
