# Supplementary material for: Intraoperative Guidance of Pancreatic Cancer Resection Using a Toll-like Receptor 2–Targeted Fluorescence Molecular Imaging Agent
Source: Cancer Res Commun. 2024 Nov 5;4(11):2877–87. doi: 10.1158/2767-9764.CRC-24-0244 (PMC11536076; doi:10.1158/2767-9764.CRC-24-0244)
Supplement: Figure S1 — Flow chart for the tumor-free survival with TLR2 targeted fluorescence-guided surgery study. [file crc-24-0244_figure_s1_suppsf1.docx]

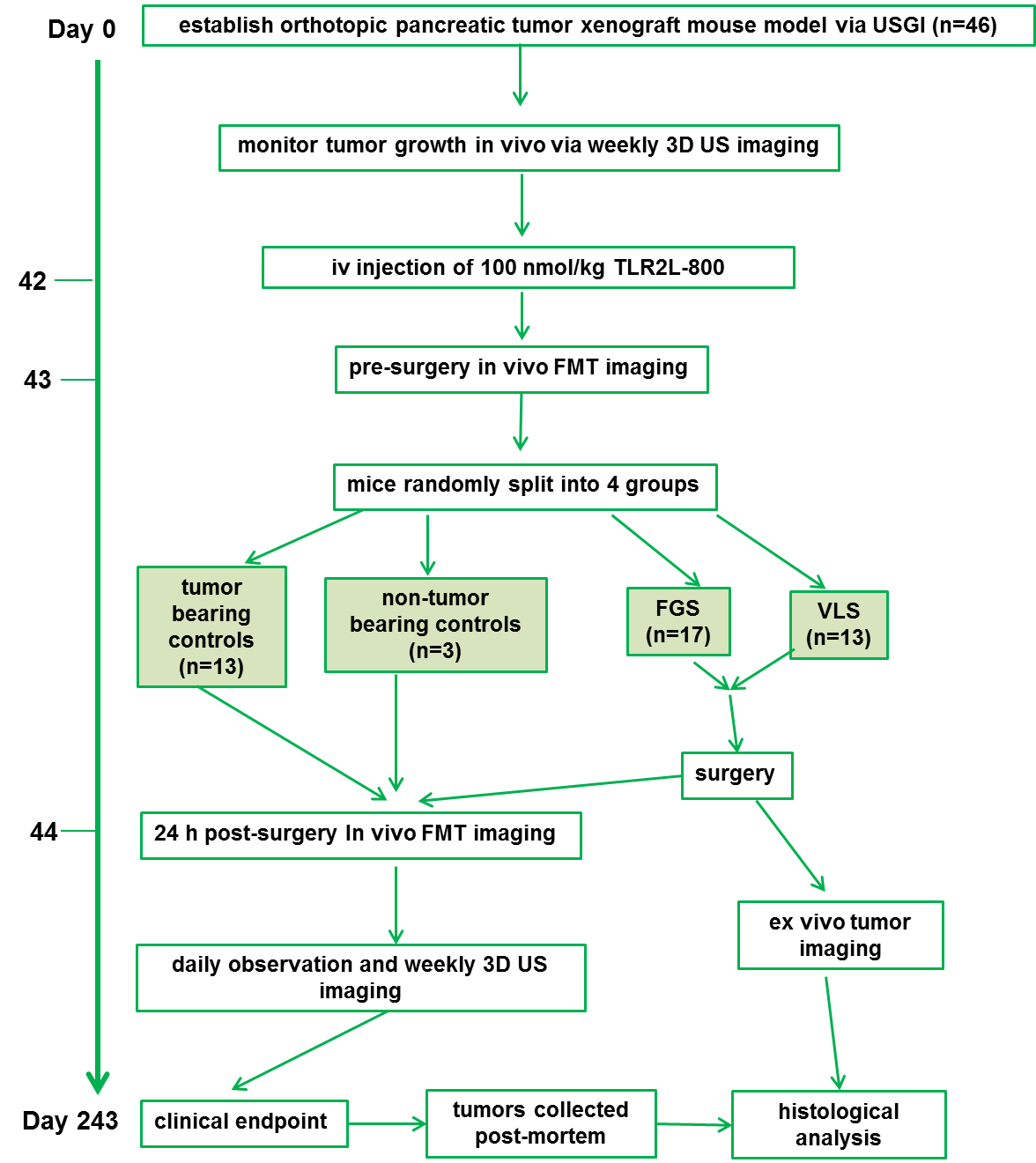


Supplementary Figure S1

**Fig. S1. Flow chart for the tumor-free survival with TLR2 targeted fluorescence-guided surgery study.** Flow chart shows the procedural steps performed for each of the 4 groups compared in the study.
