# Supplementary material for: Intraoperative Guidance of Pancreatic Cancer Resection Using a Toll-like Receptor 2–Targeted Fluorescence Molecular Imaging Agent
Source: Cancer Res Commun. 2024 Nov 5;4(11):2877–87. doi: 10.1158/2767-9764.CRC-24-0244 (PMC11536076; doi:10.1158/2767-9764.CRC-24-0244)

**Fig. S2. In vivo fluorescence-guided surgical imaging systems.** Panel (**A**) shows the preclinical system: an adapted version of Diagnostic Instruments LightTools system, outfitted with an 800 nm filter set, cooled CCD camera and SPOT Advanced imaging software. A surgical resection performed by an animal surgeon is shown with the real-time fluorescence acquired image showing the fluorescently-labeled pancreatic tumor (yellow arrow). (**B**) Shows a surgery underway using an intraoperative open air fluorescence instrument, SurgVision T3 Imaging platform.


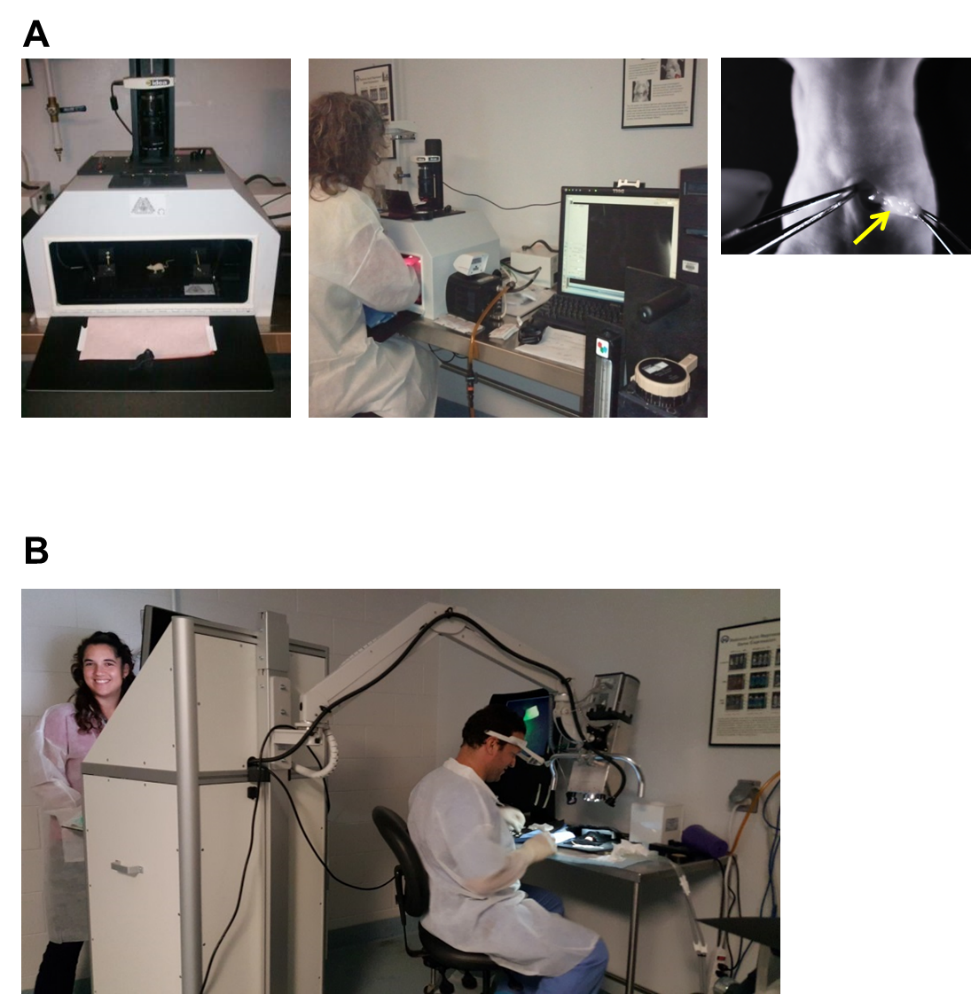


Supplementary Figure S2


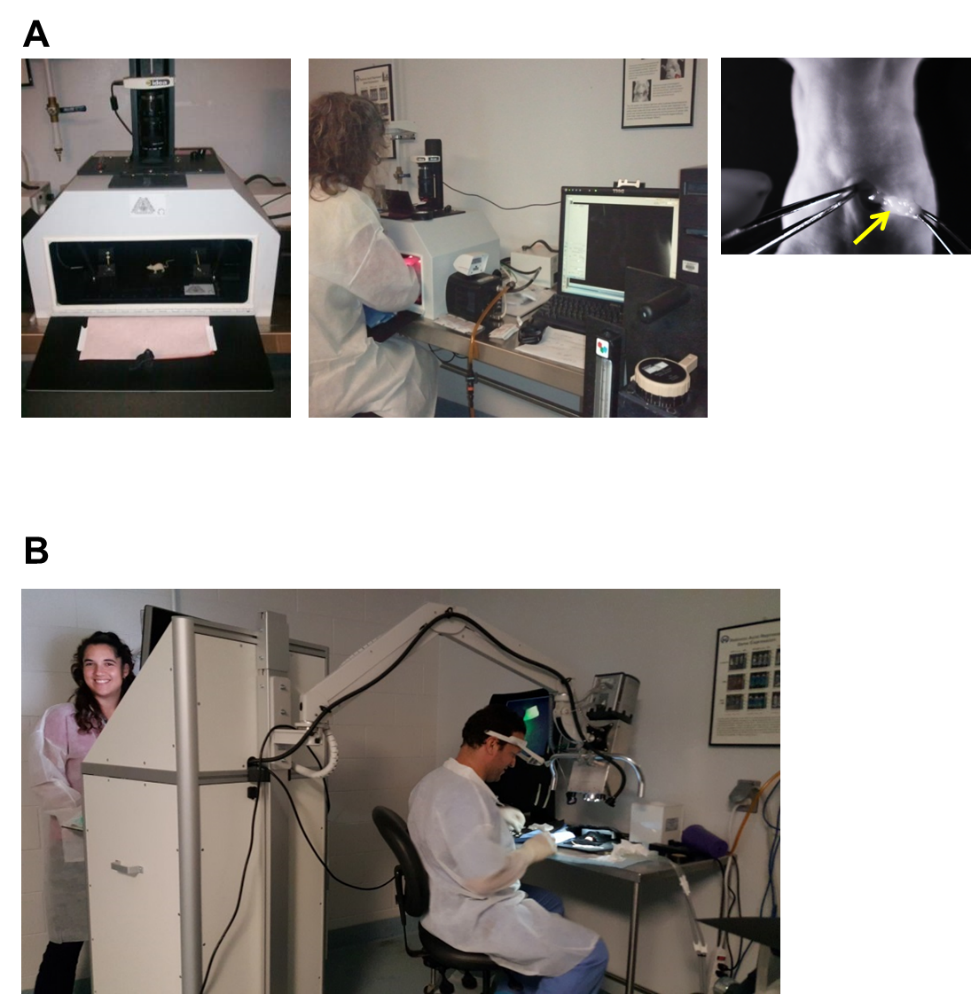

Supplement: Figure S2 — In vivo fluorescence-guided surgical imaging systems. [file crc-24-0244_figure_s2_suppsf2.docx]
