# Supplementary material for: Intraoperative Guidance of Pancreatic Cancer Resection Using a Toll-like Receptor 2–Targeted Fluorescence Molecular Imaging Agent
Source: Cancer Res Commun. 2024 Nov 5;4(11):2877–87. doi: 10.1158/2767-9764.CRC-24-0244 (PMC11536076; doi:10.1158/2767-9764.CRC-24-0244)
Supplement: Figure S3 — Optical characterization of TLR2L-800. [file crc-24-0244_figure_s3_suppsf3.docx]

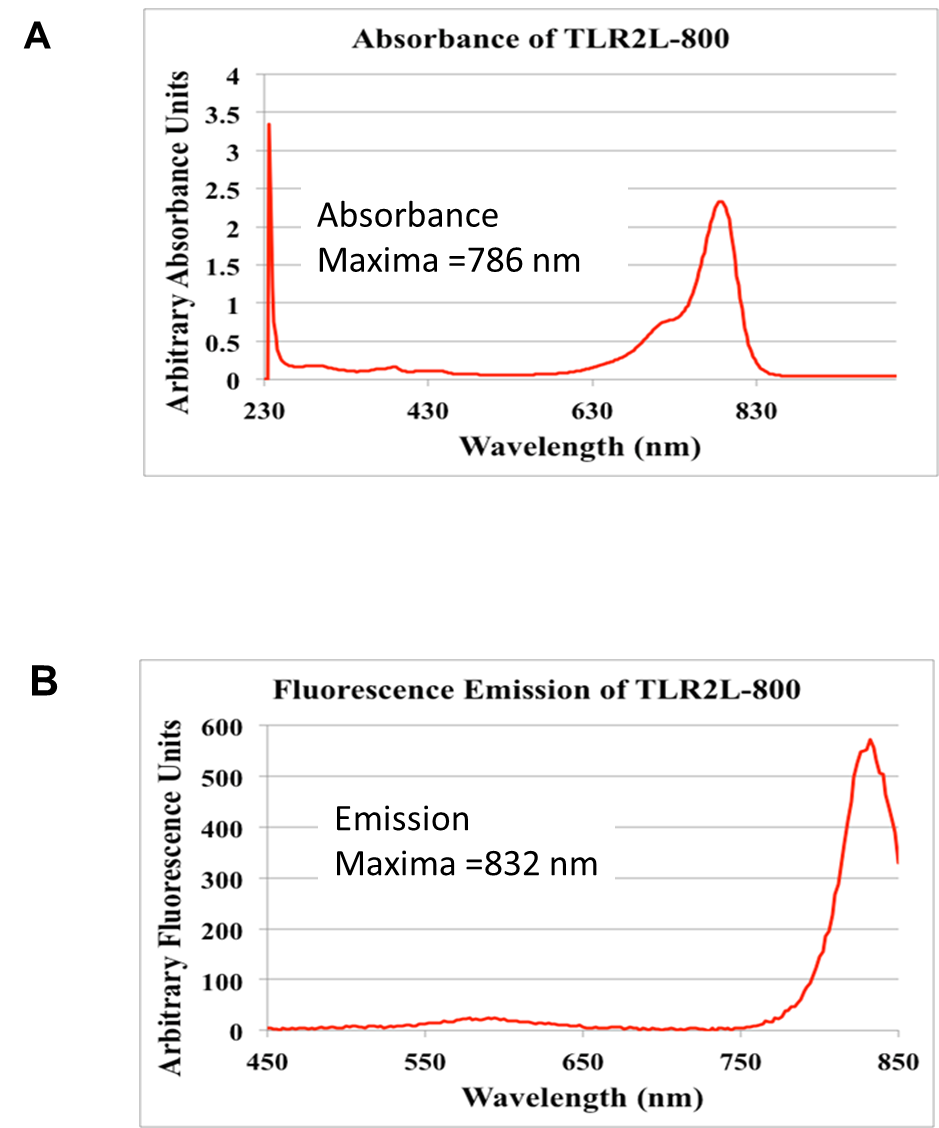


Supplementary Figure S3


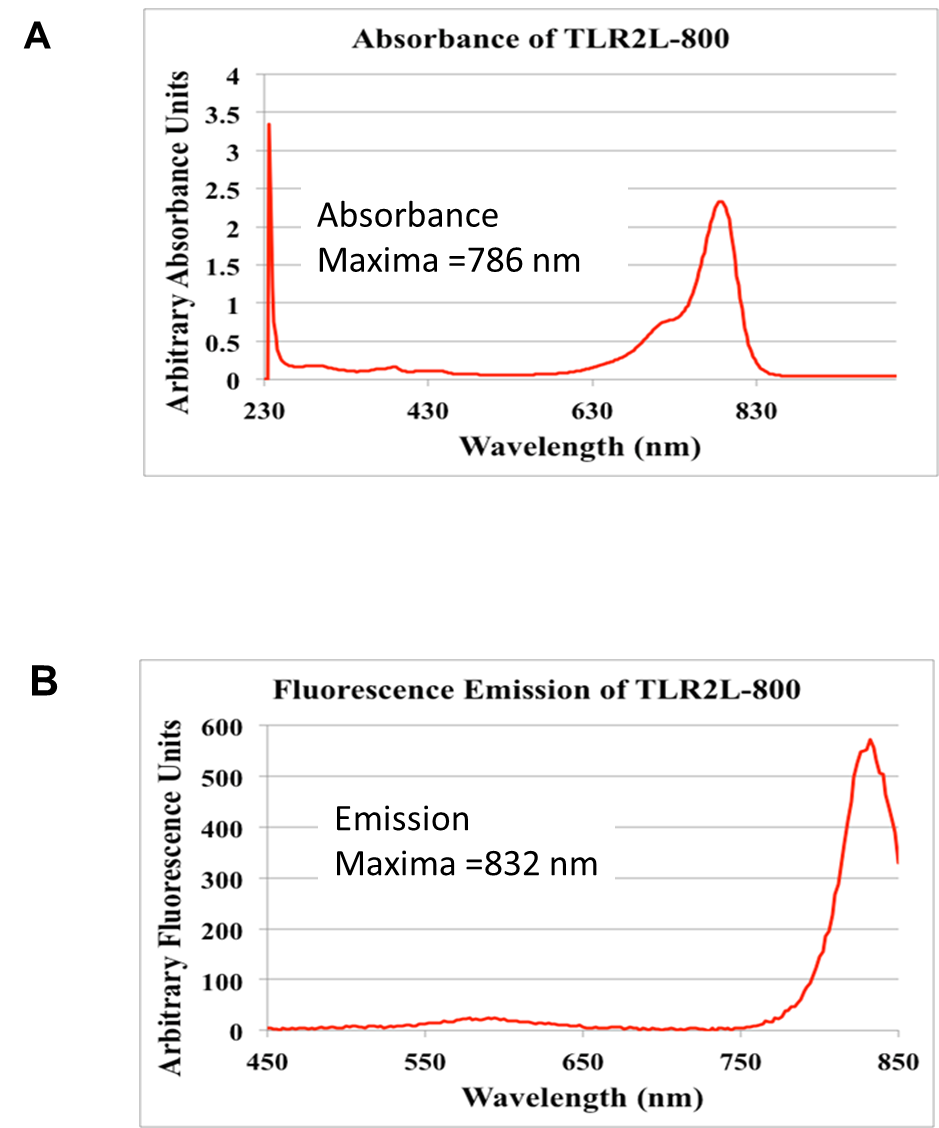


**Fig. S3. Optical characterization of TLR2L-800.** (**A**) The absorbance spectrum of TLR2L-800 is shown with an absorbance maxima of 786 nm. (**B**) The fluorescence emission spectrum is shown for TLR2L-800 with an emission maxima of 832 nm.
