# Supplementary material for: Intraoperative Guidance of Pancreatic Cancer Resection Using a Toll-like Receptor 2–Targeted Fluorescence Molecular Imaging Agent
Source: Cancer Res Commun. 2024 Nov 5;4(11):2877–87. doi: 10.1158/2767-9764.CRC-24-0244 (PMC11536076; doi:10.1158/2767-9764.CRC-24-0244)
Supplement: Figure S5 — Ex vivo biodistribution of 100 nmol/kg TLR2L-800 in mice bearing subcutaneous TLR2+ (SU.86.86) pancreatic tumor xenografts on the right flank. [file crc-24-0244_figure_s5_suppsf5.docx]

**
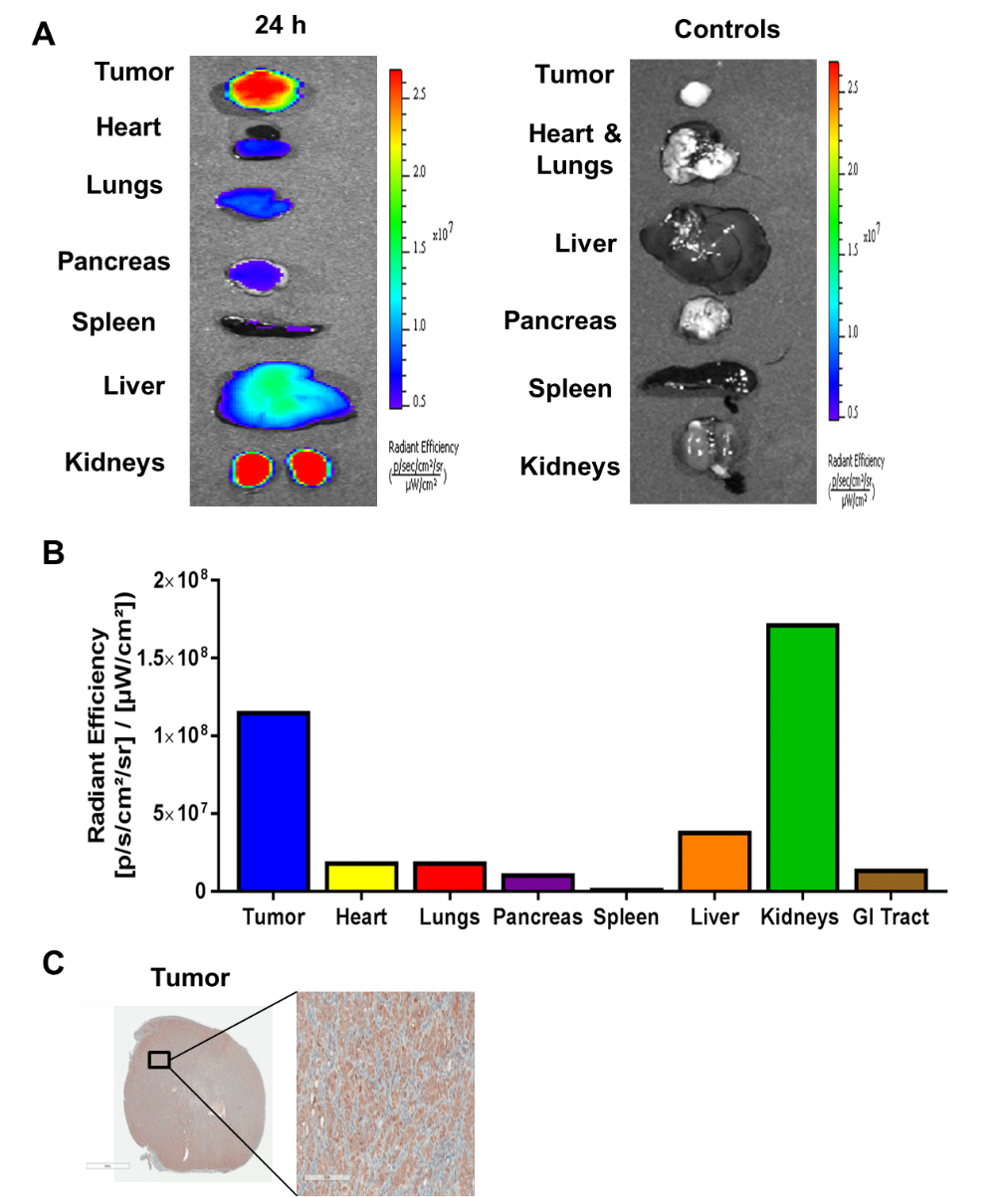
**

**Fig. S5. Ex vivo biodistribution of 100 nmol/kg TLR2L-800 in mice bearing subcutaneous TLR2+ (SU.86.86) pancreatic tumor xenografts on the right flank.** (**A**) Ex vivo fluorescence images of tumor and major organs at 24 h (left) and the corresponding non-TLR2-800-agent injected tumor-bearing no surgery mouse (right) (note both panels use the same scale). (**B**) Graph depicts the quantified ex vivo fluorescence signal obtained in the tumor and major organs at 24 h, signals were normalized to the non-TLR2-800-agent injected no surgery controls (n=1). (**C**) The corresponding IHC staining for TLR2 confirms the expression of TLR2 in the pancreatic tumor xenograft of the non-TLR2-800-agent-injected tumor-bearing no surgery mouse.
