# Supplementary material for: Intraoperative Guidance of Pancreatic Cancer Resection Using a Toll-like Receptor 2–Targeted Fluorescence Molecular Imaging Agent
Source: Cancer Res Commun. 2024 Nov 5;4(11):2877–87. doi: 10.1158/2767-9764.CRC-24-0244 (PMC11536076; doi:10.1158/2767-9764.CRC-24-0244)
Supplement: Figure S6 — Pharmacokinetics and biodistribution of 100 nmol/kg TLR2L-800 in an SU.86.86 orthotopic human pancreatic cancer xenograft mouse model. [file crc-24-0244_figure_s6_suppsf6.docx]

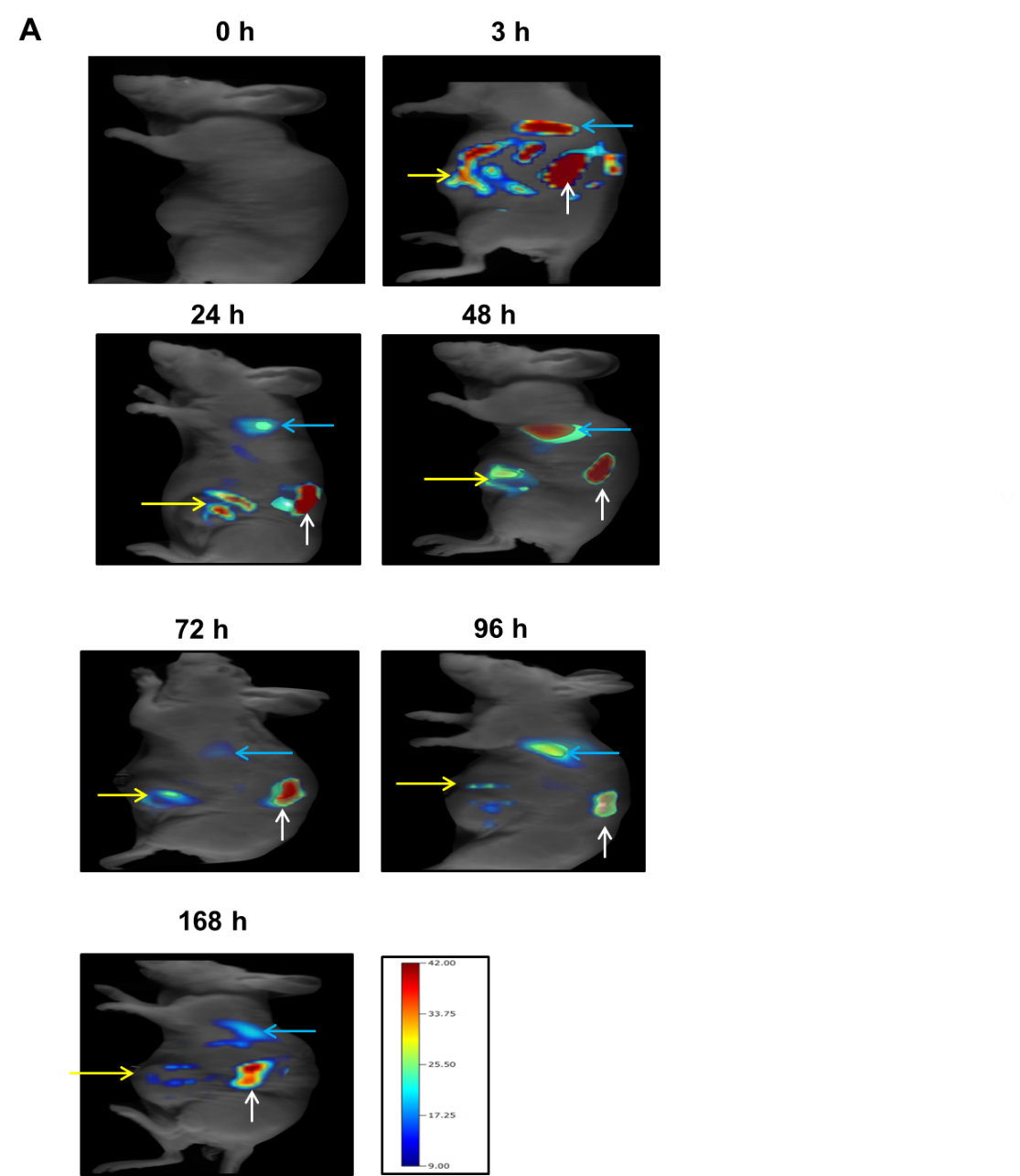


Supplementary Figure S6

**Fig. S6. Pharmacokinetics and biodistribution of 100 nmol/kg TLR2L-800 in an SU.86.86 orthotopic human pancreatic cancer xenograft mouse model. (A)** Fluorescence molecular tomography images tracking the fluorescence signal in the pancreatic tumors (yellow arrows), kidneys (white arrows) and liver (blue arrows) over time from 0 to 168 h.
