# Supplementary material for: Intraoperative Guidance of Pancreatic Cancer Resection Using a Toll-like Receptor 2–Targeted Fluorescence Molecular Imaging Agent
Source: Cancer Res Commun. 2024 Nov 5;4(11):2877–87. doi: 10.1158/2767-9764.CRC-24-0244 (PMC11536076; doi:10.1158/2767-9764.CRC-24-0244)
Supplement: Figure S7 — In vivo fluorescence molecular tomographic images of TLR2L-800 labeled SU.86.86 orthotopic pancreatic tumors in nude mice 24 h post-surgery. [file crc-24-0244_figure_s7_suppsf7.docx]

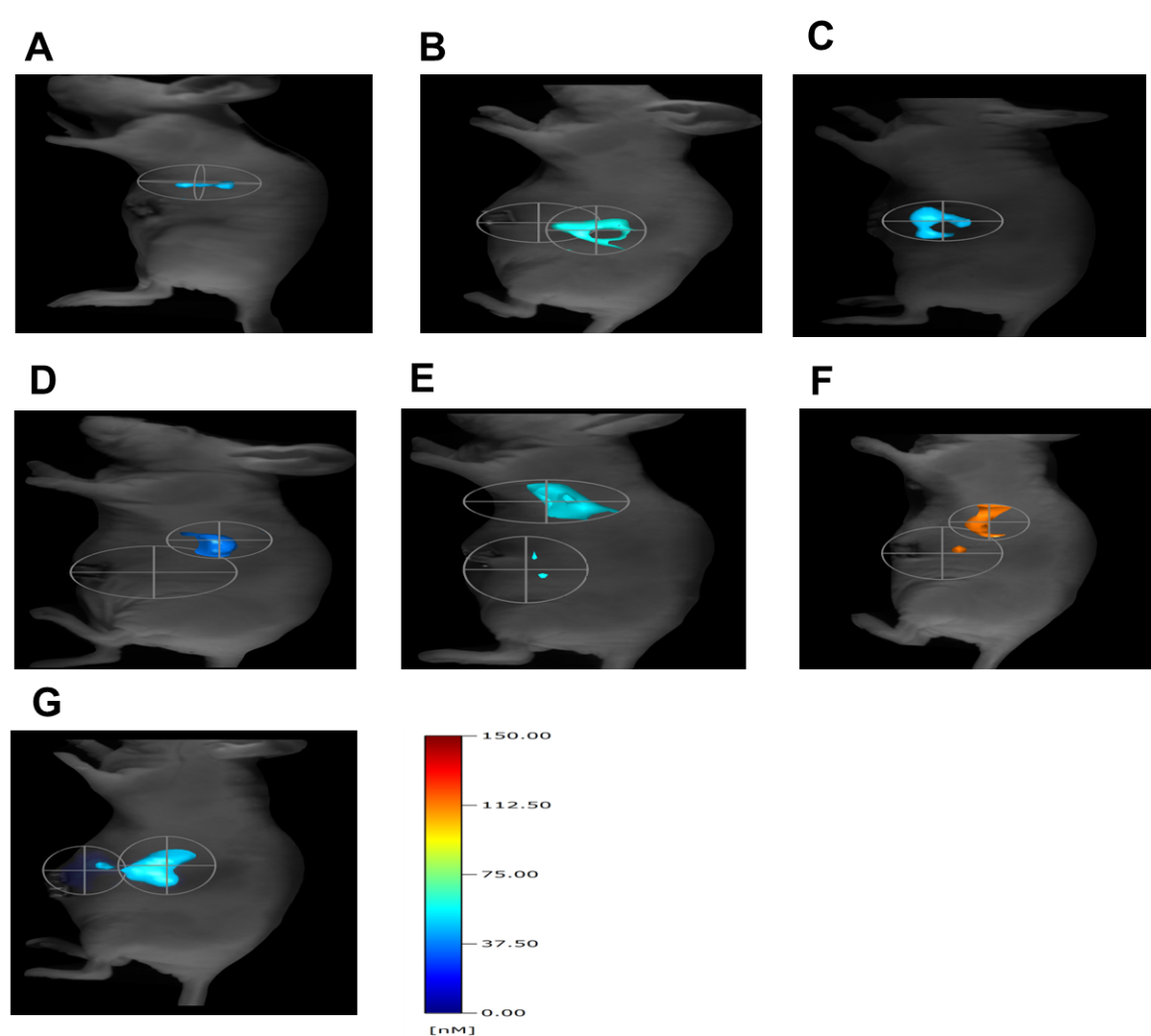


Supplementary Figure S7

**Fig. S7. In vivo fluorescence molecular tomographic images of TLR2L-800 labeled SU.86.86 orthotopic pancreatic tumors in nude mice 24 h post-surgery.** (**A**-**D**) Mice from the fluorescence-guided surgery cohorts do not show pancreatic tumors in the ROI drawn but do show tumors in other ROIs: (**A**) liver, (**B**) bladder (**C** and **D**) abdominal; (**E**) another mouse from the fluorescence-guided surgery shows pancreatic tumors and an axillary lymph node tumor. (**F**-**G**) Mice from the visible light surgery cohorts show small pancreatic tumors in the ROI drawn, and they also show other tumors in the abdominal region.
