# Supplementary material for: Intraoperative Guidance of Pancreatic Cancer Resection Using a Toll-like Receptor 2–Targeted Fluorescence Molecular Imaging Agent
Source: Cancer Res Commun. 2024 Nov 5;4(11):2877–87. doi: 10.1158/2767-9764.CRC-24-0244 (PMC11536076; doi:10.1158/2767-9764.CRC-24-0244)
Supplement: Figure S4 — In vitro TLR2-negative agonist activity control. [file crc-24-0244_figure_s4_suppsf4.docx]

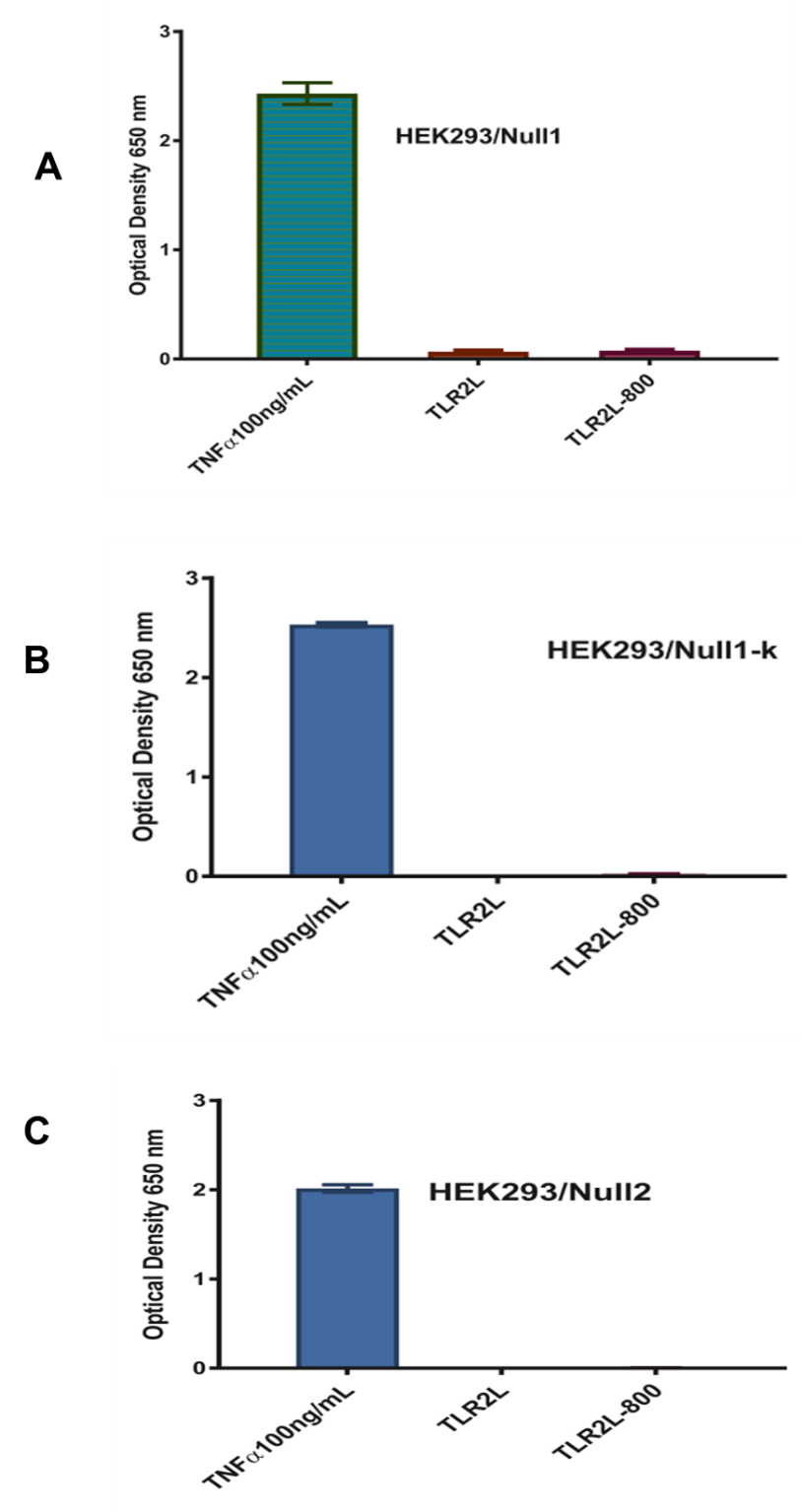


Supplementary Figure S4


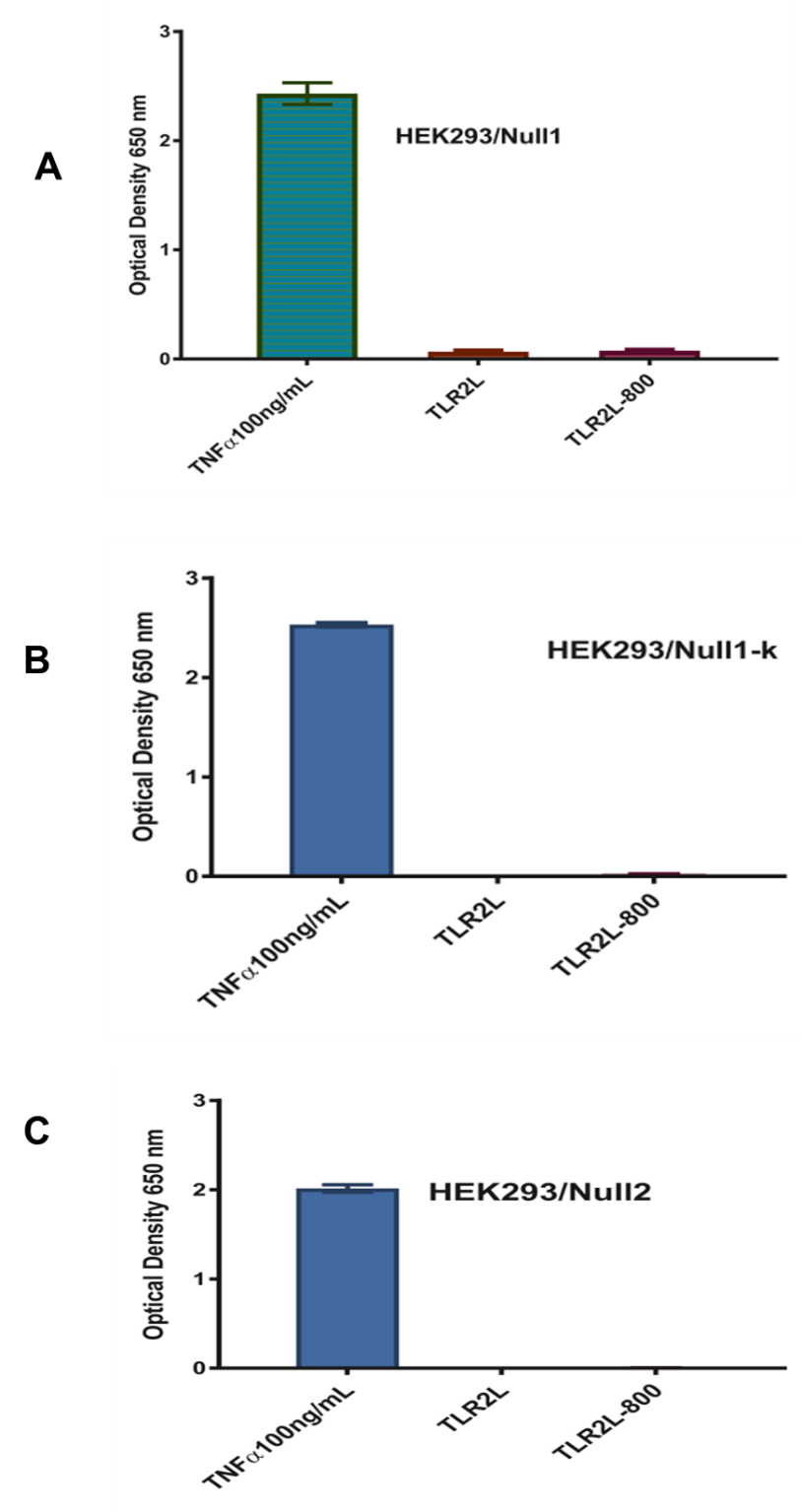

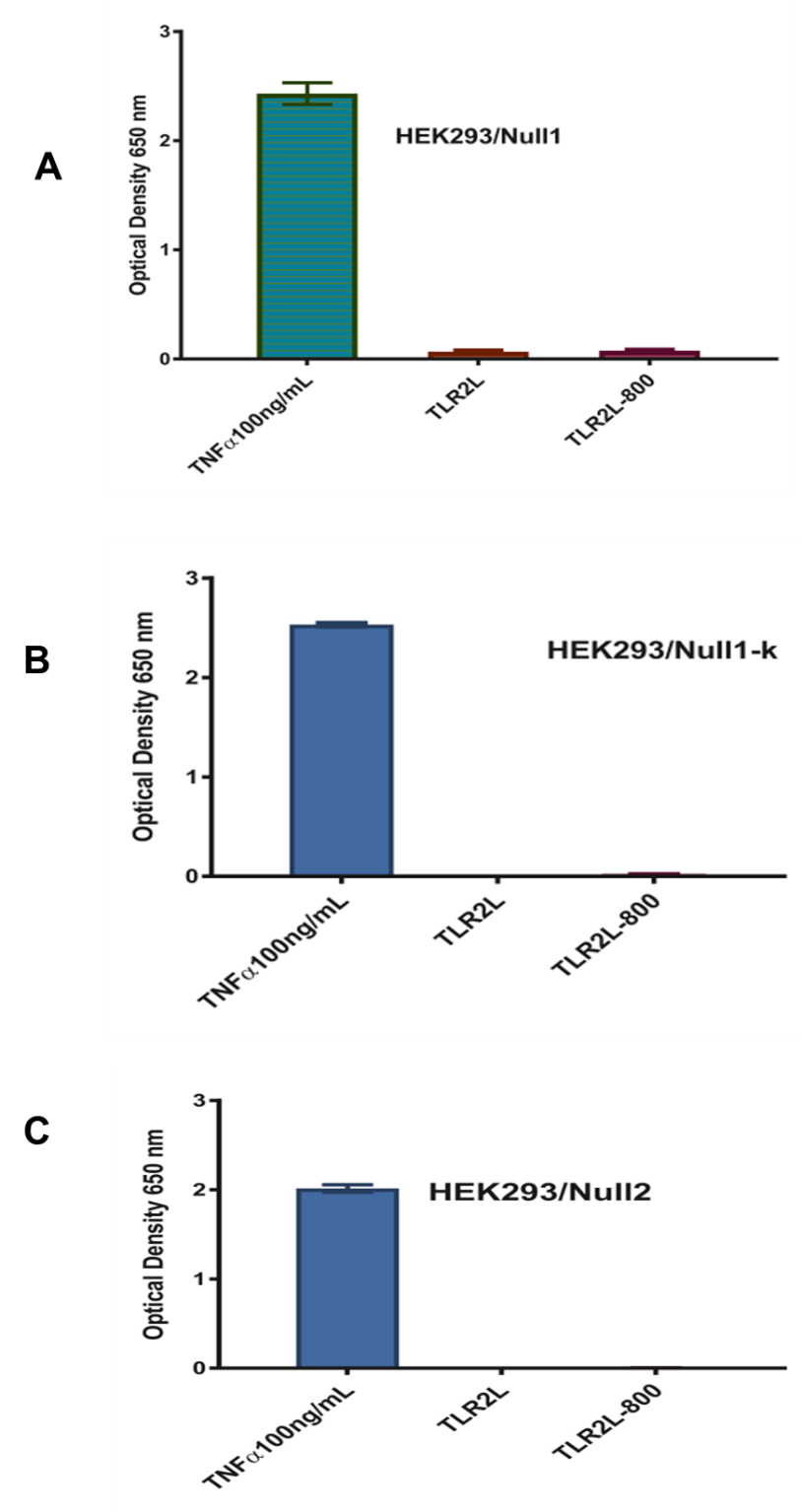


**Fig. S4. In vitro TLR2-negative agonist activity control.** Graphs show the assay results for the following TLR2-negative cells lines: (**A**) HEK293/Null1, (**B**) HEK293/Nullk and (**C**) HEK293/Null2. TNFα is a positive control that activates NF-κB signaling independent of TLR2. Results shown are the mean of 3 independent assays performed in triplicate wells.
