# Supplementary material for: Intraoperative Guidance of Pancreatic Cancer Resection Using a Toll-like Receptor 2–Targeted Fluorescence Molecular Imaging Agent
Source: Cancer Res Commun. 2024 Nov 5;4(11):2877–87. doi: 10.1158/2767-9764.CRC-24-0244 (PMC11536076; doi:10.1158/2767-9764.CRC-24-0244)
Supplement: Table S1 — Expression of pancreatic tumors identified by various methods for the 4 survival study cohorts. [file crc-24-0244_table_s1_suppst1.docx]

**Table S1.** Expression of pancreatic tumors identified by various methods for the 4 survival study cohorts.

|  |  | Pancreatic Tumor Present (%) | | |
| --- | --- | --- | --- | --- |
| Type | Cohort Size (n) | FMT 24 h  Post-surgery | Visual Exam Post-Mortem | H&E Stain |
| Non-tumor-bearing no surgery | 3 | 0 | 0 | 0 |
| Tumor-bearing no surgery | 13 | 100 | 100 | 100 |
| Visible light surgery | 13 | 100 | 69 | 100 |
| Fluorescence-guided surgery | 17 | 59 | 24 | 41 |
