# Supplementary material for: Intraoperative Guidance of Pancreatic Cancer Resection Using a Toll-like Receptor 2–Targeted Fluorescence Molecular Imaging Agent
Source: Cancer Res Commun. 2024 Nov 5;4(11):2877–87. doi: 10.1158/2767-9764.CRC-24-0244 (PMC11536076; doi:10.1158/2767-9764.CRC-24-0244)
Supplement: Table S2 — Identification of pancreatic tumors and suspected cause of death for individual mice. [file crc-24-0244_table_s2_suppst2.docx]

**Table S2.** Identification of pancreatic tumors and suspected cause of death for individual mice.

|  | | | Pancreatic Tumor Present (Y/N) | | | |  |
| --- | --- | --- | --- | --- | --- | --- | --- |
| **Experiment** | **Mouse #** | **Survival**  **(Days)** | **Visual Exam**  **Post-Mortem** | **H&E**  **Stain** | **TLR2 IHC**  **Stain** | **FMT 24 h**  **Post-Surgery** | **Suspected Cause of Death** |
| Non-tumor bearing controls (n=3) | 1 | 200 | N | N | N | N | study end point |
|  | 3 | 200 | N | N | N | N | study end point |
|  | 37 | 200 | N | N | N | N | study end point |
| Tumor bearing controls (n=13) | 7 | 17 | Y | Y | Y | Y | pancreatic tumor |
|  | 10 | 41 | Y | Y | Y | Y | pancreatic tumor |
|  | 11 | 16 | Y | Y | Y | Y | pancreatic tumor |
|  | 12 | 12 | Y | Y | Y | n/a | pancreatic tumor |
|  | 13 | 29 | Y | Y | Y | n/a | pancreatic tumor |
|  | 14 | 43 | Y | Y | Y | n/a | pancreatic tumor |
|  | 16 | 17 | Y | Y | Y | n/a | pancreatic tumor |
|  | 24 | 16 | Y | Y | Y | n/a | pancreatic tumor |
|  | 25 | 16 | Y | Y | Y | n/a | pancreatic tumor |
|  | 26 | 16 | Y | Y | Y | n/a | pancreatic tumor |
|  | 35 | 23 | Y | Y | Y | n/a | pancreatic tumor |
|  | 36 | 29 | Y | Y | Y | n/a | pancreatic tumor |
|  | 38 | 47 | Y | Y | Y | n/a | pancreatic tumor |
| Visible light surgery (n=13) | 11 | 41 | Y | Y | Y | Y | pancreatic tumor |
|  | 15 | 23 | Y | Y | Y | Y | pancreatic tumor |
|  | 19 | 23 | Y | Y | Y | Y | pancreatic tumor |
|  | 21 | 27 | Y | Y | Y | Y | pancreatic tumor |
|  | 23 | 27 | Y | Y | Y | Y | pancreatic tumor |
|  | 27 | 20 | N | Y | Y | Y | abdominal tumor |
|  | 29 | 41 | Y | Y | Y | Y | pancreatic tumor |
|  | 31 | 27 | N | Y | Y | Y | abdominal tumor |
|  | 33 | 27 | Y | Y | Y | Y | pancreatic tumor |
|  | 39 | 23 | Y | Y | Y | Y | pancreatic tumor |
|  | 41 | 20 | Y | Y | Y | Y | pancreatic tumor |
|  | 43 | 20 | Y | Y | Y | Y | pancreatic tumor |
|  | 45 | 29 | Y | Y | Y | Y | pancreatic tumor |
| Fluorescence guided surgery (n=17) | 2 | 200 | N | N | N | N | study end point |
|  | 4 | 200 | N | N | N | N | study end point |
|  | 6 | 200 | N | N | N | N | study end point |
|  | 8 | 28 | N | N | N | Y | liver tumor |
|  | 12 | 200 | N | N | N | N | study end point |
|  | 16 | 15 | N | N | N | N | bladder tumor |
|  | 18 | 200 | N | N | N | N | study end point |
|  | 20 | 23 | N | Y | Y | Y | axillary lymph node tumor |
|  | 22 | 43 | N | Y | Y | Y | pancreatic tumor |
|  | 28 | 35 | N | Y | Y | Y | pancreatic tumor |
|  | 30 | 200 | N | N | N | N | study end point |
|  | 32 | 47 | Y | Y | Y | Y | subcutaneous tumor |
|  | 34 | 29 | Y | Y | Y | Y | pancreatic tumor |
|  | 40 | 16 | N | N | N | N | abdominal tumor |
|  | 42 | 35 | N | Y | Y | Y | abdominal tumor |
|  | 44 | 16 | N | N | N | N | abdominal tumor |
|  | 46 | 47 | Y | Y | Y | Y | subcutaneous tumor |
